# Ionic Route to Atmospheric Relevant HO_2_ and Protonated Formaldehyde from Methanol Cation and O_2_

**DOI:** 10.3390/molecules29071484

**Published:** 2024-03-27

**Authors:** Mauro Satta, Daniele Catone, Mattea Carmen Castrovilli, Francesca Nicolanti, Antonella Cartoni

**Affiliations:** 1 Institute for the Study of Nanostructured Materials-CNR (ISMN-CNR), Department of Chemistry, Sapienza University of Rome, P. le Aldo Moro 5, 00185 Rome, Italy; 2 Istituto di Struttura della Materia-CNR (ISM-CNR), Area della Ricerca di Roma 2, Via del Fosso del Cavaliere 100, 00133 Rome, Italy; daniele.catone@cnr.it; 3 Istituto di Struttura della Materia-CNR (ISM-CNR), Area della Ricerca di Roma 1, 00015 Rome, Italy; matteacarmen.castrovilli@cnr.it; 4 Department of Physics, Sapienza University of Rome, P. le Aldo Moro 5, 00185 Rome, Italy; francesca.nicolanti@uniroma1.it; 5 Department of Chemistry, Sapienza University of Rome, P. le Aldo Moro 5, 00185 Rome, Italy

**Keywords:** rate coefficient, cosmic rays, hydrogen transfer, ion molecule reaction, synchrotron radiation, ab initio calculations

## Abstract

Gas-phase ion chemistry influences atmospheric processes, particularly in the formation of cloud condensation nuclei by producing ionic and neutral species in the upper troposphere–stratosphere region impacted by cosmic rays. This work investigates an exothermic ionic route to the formation of hydroperoxyl radical (HO_2_) and protonated formaldehyde from methanol radical cation and molecular oxygen. Methanol, a key atmospheric component, contributes to global emissions and participates in various chemical reactions affecting atmospheric composition. The two reactant species are of fundamental interest due to their role in atmospheric photochemical reactions, and HO_2_ is also notable for its production during lightning events. Our experimental investigations using synchrotron radiation reveal a fast hydrogen transfer from the methyl group of methanol to oxygen, leading to the formation of CH_2_OH^+^ and HO_2_. Computational analysis corroborates the experimental findings, elucidating the reaction dynamics and hydrogen transfer pathway. The rate coefficients are obtained from experimental data and shows that this reaction is fast and governed by capture theory. Our study contributes to a deeper understanding of atmospheric processes and highlights the role of ion-driven reactions in atmospheric chemistry.

## 1. Introduction

Gas-phase ion chemistry is a pivotal aspect of atmospheric processes, exerting a significant influence on the reactivity of various species. Despite the crucial role of neutrals in chemical processes, ions display remarkable reactivity, often surpassing neutral–neutral reactions by up to 10 orders of magnitude [1]. This heightened reactivity extends to the formation of cloud condensation nuclei (CCN), where ions act as primary triggers for cloud formation [2]. The atmospheric relevance of ionic processes is further emphasized in the upper troposphere and stratosphere, where the ionization caused by cosmic rays’ energetic subatomic particles entering the Earth’s atmosphere plays a crucial role [3]. Despite recent advancements, the comprehensive understanding of the fundamental role of ions in atmospheric and climate models remains challenging, with their consideration in predictive models limited, necessitating further exploration for an enhanced understanding.

This topic has garnered considerable attention, leading to the initiation of the Cosmics Leaving Outdoor Droplets (CLOUD) experiment at CERN in 2006. The experiment aimed to explore the influence of galactic cosmic rays (GCRs) on aerosols, cloud formation, and their implications for the climate [4]. Understanding the role of trace gases, including carbon dioxide, methane, water, nitrous oxide, ozone, and sulfur dioxide, in aerosol particle formation linked to ions is crucial for comprehending atmospheric chemical networks. These gases, emitted by natural or anthropogenic sources, contribute to chemical reactions altering the budget of climate-relevant species, impacting atmospheric composition [5].

The intricate relationship between ions, aerosols, clouds, and their connection to global warming and climate change remains a subject of controversy, with ongoing debates fueled by laboratory measurements, satellite data analysis, and climate models [6]. Our group has dedicated efforts to explore the reactivity of ionized trace gases, such as CO_2_, SO_2_, CH_4_, N_2_O, and CH_3_OH, through theoretical and experimental studies [7,8,9,10,11]. This involves employing tunable synchrotron radiation with neutral species like molecular hydrogen and water.

In this context, our research delves into the study of the reaction of the methanol cation with molecular oxygen, a topic that, to our knowledge, has not been previously studied. Methanol plays a fundamental role in atmospheric chemistry [12], primarily sourced from terrestrial plants [13], contributing to a significant global emission estimated at 70–350 Tg methanol/year [14]. Approximately 20% of total global volatile organic compound (VOC) emissions come from methanol, with only methane having a greater abundance [15]. Primary methanol sinks include surface deposition, ocean uptake, and reactions with OH radicals [16,17,18]. Furthermore, tropospheric O_3_, OH, CO, and formaldehyde chemistry is intricately linked with methanol photochemistry [19].

Observations of methanol in the gas phase extend beyond Earth, with sightings in various interstellar environments such as hot cores, dark clouds, and diffuse molecular gas [20,21,22,23]. Methanol has been the subject of thermal and photochemical studies for neutral CH_3_OH and CH_3_OH–O_2_ on astrophysical ices [24].

The methanol cation can react with molecular oxygen according to the following reactions (the formation enthalpy are from NIST’s data [25]):

$$CH_{3}OH^{•+}+O_{2}\to \left\{\begin{array}{cc} CH_{2}OH^{+}+HO_{2} & \Delta_{f}H^{\circ }=-118.4\;\mathrm{kJ}/\mathrm{mol}\\ CH_{3}O^{+}+HO_{2} & \Delta_{f}H^{\circ }=+212.3\;\mathrm{kJ}/\mathrm{mol} \end{array}\right.$$

The first exothermic reaction is noteworthy not only for the nature of the reactants, which play a prominent role in atmospheric chemistry, but also for both the neutral and ionic products—HO_2_ and the protonated formaldehyde—generated in this reaction. Two other less exothermic channels can be predicted by thermochemical data leading to $H_{2}CO+H_{2}O_{2}^{+}$ and $H_{2}CO^{+}+H_{2}O_{2}$ with $\Delta_{f}H^{\circ }=-72.0\;\mathrm{kJ}/\mathrm{mol}$ and $\Delta_{f}H^{\circ }=-43.1\;\mathrm{kJ}/\mathrm{mol}$, respectively [25]. However, these two channels have not been observed under the experimental conditions used in this work.

The protonated formaldehyde can also lead to the formation of formaldehyde through fast proton transfer processes with molecules having greater proton affinity, such as methanol and ammonia [25]. Formaldehyde is an important trace gas present in the troposphere that can alter the molecular budget of substances like ozone and also HO_2_. It participates in complex photochemical processes that lead to the production of atmospheric pollutants, contributes to particulate matter formation, and it is a precursor of chemical species which determine the acidity of clouds and rainwater [26,27,28].

The hydroperoxyl radical (HO_2_), a significant free radical in the atmosphere, participates in photochemical reactions that influence natural and anthropogenic emissions. Notably, HO_2_ reactions with Criegee intermediates play a crucial role in tropospheric chemistry [29]. The production of hydroperoxyl radicals during lightning events has been studied, and its occurrence in spatially and temporally localized conditions of high ionization links the production of HO_2_ to ion reactivity [30]. The kinetics of the reaction $HO_{2}+HO_{2}\to H_{2}O_{2}+O_{2}$ and its implications for stratospheric H_2_O_2_ have also been investigated [31]. Moreover, HO_2_’s role in urban smog formation, particularly its reaction with NO to produce NO_2_ and generate O_3_ after photofragmentation in the troposphere, adds another layer to its atmospheric significance [32]. Furthermore, the hydroperoxyl radical is involved in the formation of HIO_2_ from iodine dioxide (OIO) in the atmosphere above marine and polar regions, where HIO_2_ plays a major role as a nucleating vapor with H_2_SO_4_ [33].

Our exploration of the ionic route to hydroperoxyl radical formation from methanol cation and molecular oxygen contributes to a broader understanding of atmospheric processes, emphasizing the necessity for a comprehensive consideration of ions in climate models.

This work is organized as follows. Firstly, the Section 2 provides the experimental data and the theoretical calculations with a complete picture of the mechanism of the reaction. Subsequently, details about experimental and theoretical methodologies are given in the Section 3, and a Section 4 summarizes the most significant results of this work. The dot in the CH_3_OH^•+^ is omitted in the following for the sake of clarity.

## 2. Results and Discussions

**Figure 1:**
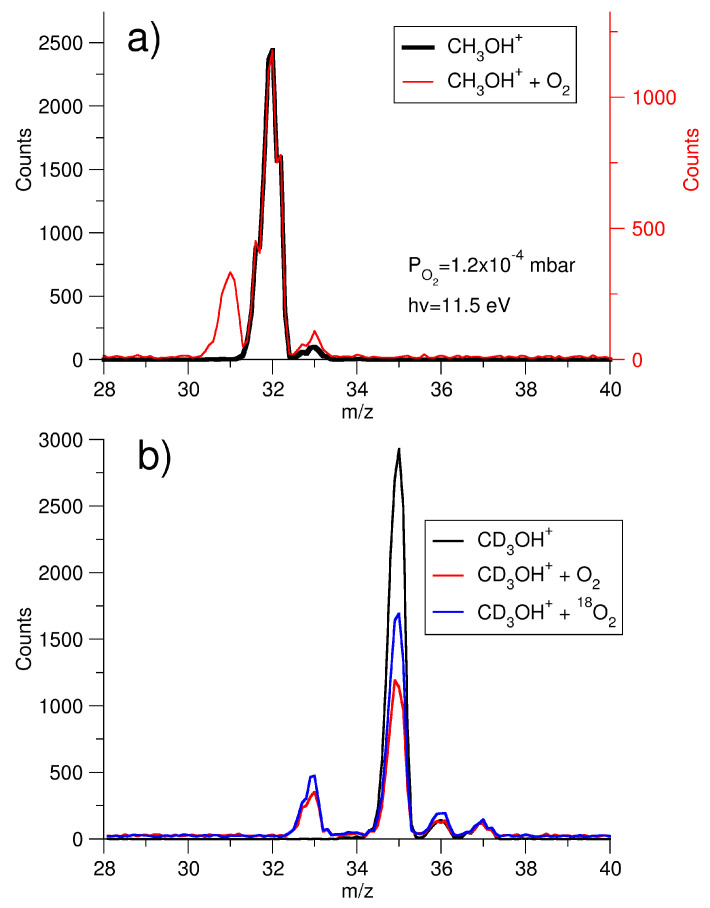
(**a**) Mass/charge (*m*/*z*) spectra of the ion CH_3_OH^+^, acquired at the photon energy of 11.5 eV without (black line) and with O_2_ (red line) in the octupole at the oxygen pressure of 1.2 × 10^−4^ mbar and CE = 0. (**b**) mass/charge (*m*/*z*) spectra of the ion CD_3_OH^+^, acquired at the photon energy of 11.5 eV without (black line) and with O_2_ (red line) or with ^18^O_2_ (blue line) in the octupole at a pressure of about 10^−4^ mbar and CE = 0.

The reactivity of the methanol radical cation with oxygen was initially studied by reacting CH_3_OH^+^, generated in the ion source by synchrotron radiation, with O_2_ introduced into the octupole. The ion was generated within an energy range between its ionization energy of 10.84 ± 0.01 eV [25] and 11.6 eV so to avoid the effect of its fragmentation into CH_2_OH^+^ occurring at 11.649 ± 0.003 eV [34]. The mass spectrum of the ion molecule reaction, acquired with the quadrupole and shown in Figure 1a (red line) at h*ν* = 11.5 eV, at zero collision energy (CE) and at an oxygen pressure of 1.2 × 10^−4^ mbar, demonstrates the presence not only of the peak at *m*/*z* = 32 related to methanol radical cation reagent, but also a peak at *m*/*z* = 31 as the product of the reaction.

**Figure 2:**
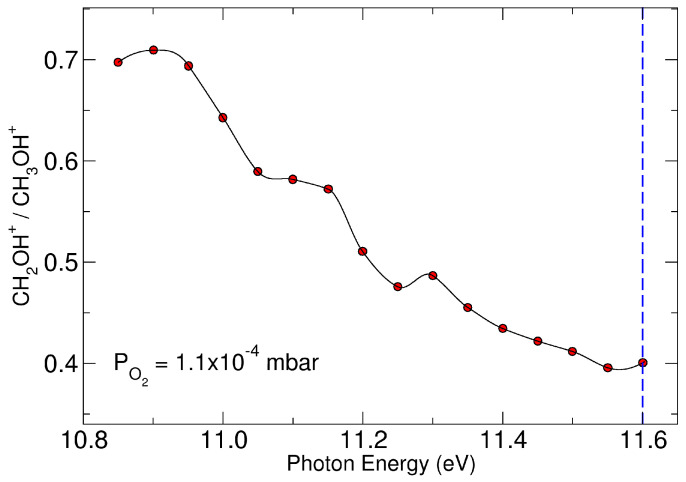
Ratio of CH_2_OH^+^ (*m*/*z* = 31) over CH_3_OH^+^ (*m*/*z* = 32) as a function of photon energy (eV) at the pressure of O_2_ of 1.1 × 10^−4^ mbar and CE = 0. A blue dashed line marks the appearance energy of CH_2_OH^+^ from CH_3_OH^+^.

Charge exchange between CH_3_OH^+^ and O_2_, leading to $O_{2}^{+}$ with a *m*/*z* ratio of 32, has been ruled out as O_2_ has a higher ionization energy 12.0697 ± 0.0002 eV [25] than methanol and the collision occurs at CE = 0. The peak observed at *m*/*z* = 33 is the protonated methanol, formed in the source after photoionization and it is also observed when there is no oxygen in the octupole (black line of Figure 1a). The ion at *m*/*z* = 31 could be CH_2_OH^+^ and/or CH_3_O^+^. To distinguish between these two isomers, experiments were conducted using deuterated methanol (CD_3_OH, 35 a.m.u.) and oxygen, both with natural abundance O_2_ and oxygen labeled with ^18^O (^18^O_2_) (Figure 1b). Mass spectra clearly show that the only observed peak resulting from the reaction is at *m*/*z* = 33 (red and blue lines in Figure 1b), while those, very tiny, at *m*/*z* = 36 and 37, are present even in the absence of oxygen (black line in Figure 1b) and are attributable to the isotopic patterns of the molecule also present in the electron impact (EI) spectrum [25] and to protonation occurring in the source after ionization, as previously observed in our study [10]. These data definitively demonstrate that the only reactive channel is the transfer of one of the deuterium of CD_3_ to oxygen, resulting in the formation of the charged species CD_2_OH^+^ (*m*/*z* = 33) and the neutral product DOO. The reactivity was also studied as a function of photon energy in the range of 10.8 < h*ν* < 11.6 eV at the oxygen pressure of 1.1 × 10^−4^ mbar and the ratio of the intensity of the product (CH_2_OH^+^ *m*/*z* = 31) vs. reagent (CH_3_OH^+^ (*m*/*z* = 32) is reported in Figure 2, where it is shown that the reactivity decreases with photon energy. The experimental results clearly demonstrate that a hydrogen transfer from C–H bond of methanol to one of the oxygen atom of O_2_ is the only observed process with an exothermicity evaluated to be 118.4 kJ/mol from literature data at 298 K [25]. This process is negatively affected by the photon energy up to h*ν* = 11.6 eV. The exclusive efficient H-transfer from the C–H bond compared to H-transfer from the O–H bond, of comparable binding energy [35], is due to the endothermicity of the process of about 212.3 kJ/mol when O–H abstraction is considered. To gain a deeper insight into reaction dynamics, theoretical calculations have been performed, considering that only one channel is experimentally observed.

**Figure 3:**
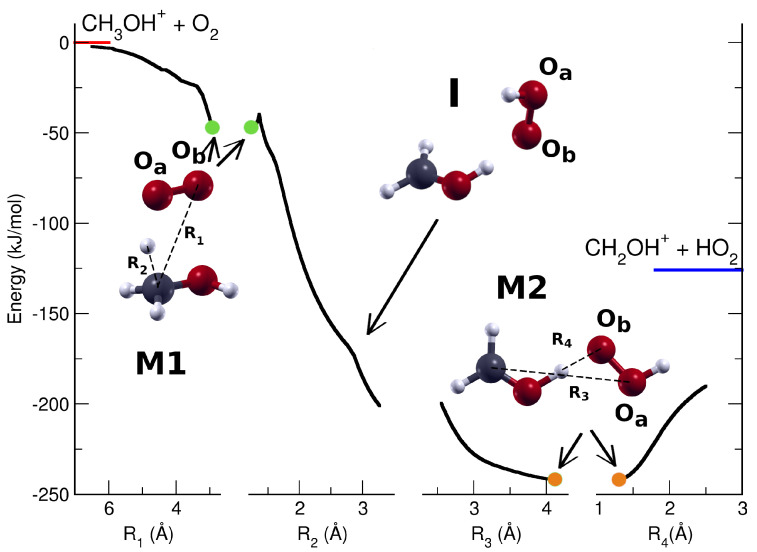
Minimum energy path of the reaction between methanol cation and molecular oxygen. The green circles indicate the position of the energy minimum M1, the orange circles indicate the position of the absolute minimum M2. The red line in the first panel is the energy reference associated with the reactive molecules, while the blue line indicates the energy of the products. See text for further details.

Two stable bimolecular adducts were calculated in the reactant region: one is characterized by a hydrogen bond between $O_{2}$ and the hydrogen atom of OH group of methanol (see M1B in Table S1 of Supplementary Materials, SM), while the other adduct M1, more stable by 30.1 kJ/mol with respect to M1B, has the $O_{2}$ parallel to the C–O bond of methanol (see M1 in Figure 3, and Table S2 of SM).

The MEP (Minimum Energy Path) of the hydrogen transfer from the methyl group of CH_3_OH^+^ to the O_2_ molecule was calculated by scanning the distance between the carbon atom and one of the oxygen atoms (see R_1_ in the inset of Figure 3) and optimizing all the other geometrical parameters. Here, the energy decreases up to −46.1 kJ/mol, where the stable bimolecular adduct M1 is formed: the interatomic distance between the O*_a_* oxygen atom and the closest hydrogen of CH_3_ is 1.39 Å, while the C-H bond (R_2_) has elongated up to 1.27 Å, and the O*_b_* oxygen of O_2_ and the O of methanol are far apart by 2.64 Å.

**Figure 4:**
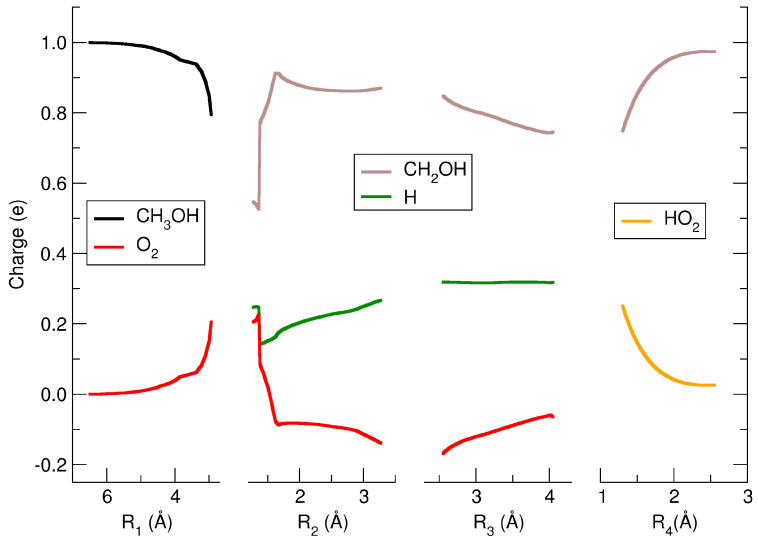
Partial charges of the reactive complex along the MEP. The H is the hydrogen transferred from the methyl group of methanol to the molecular oxygen.

**Figure 5:**
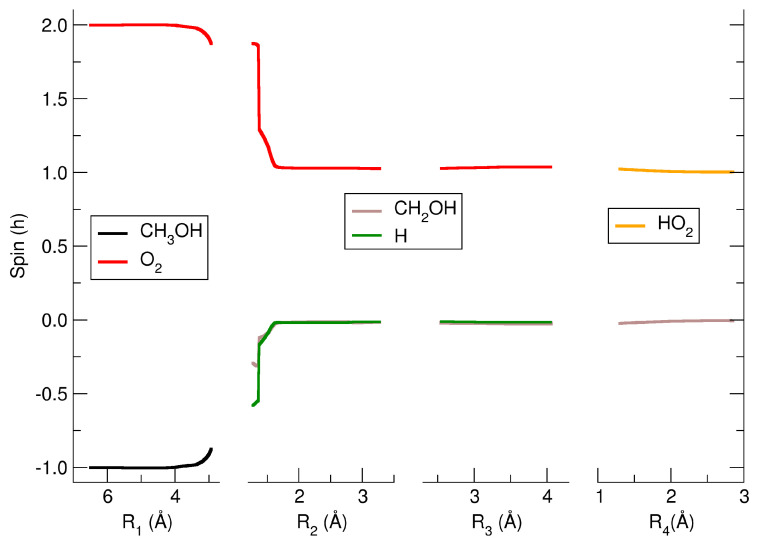
Partial spin distribution of the reactive complex along the reactive MEP.

In this adduct, there is a partial electron transfer from the O_2_ to the methanol cation (see first panel of Figure 4): about 20% of the electron charge has been moved through the CH–O hydrogen bond. The O_2_ remains almost in its triplet state (1.87*ℏ*), and the CH_3_OH^+^ is a quasi-doublet (0.87*ℏ*) as shown in the first panel of Figure 5.

The reaction proceeds afterward along the coordinate R_2_ (second panel of Figure 3), which is the C–H bond, and the MEP shows a small subreactive barrier of 6.4 kJ/mol from the first molecular adduct M1 and with R_2_ = 1.36 Å. The reactive complex along the MEP just after this barrier and H transfer, rotates (see structure I) and shows a strong charge rearrangement as can be seen in the second panel of Figure 4, where the partial charge of the CH_2_OH goes from 0.55e of the M1 to a maximum of 0.91e for R_2_ = 1.62 Å, while the molecular oxygen changes its partial charge from 0.20e to −0.08e, and the hydrogen which is transferred from the methyl group to the O_2_ varies its partial charge from +0.25e to +0.16e.

Along the R_3_ (third panel of Figure 3) coordinate which connects the carbon atom with the oxygen atom O*_a_* of HO_2_, the MEP is associated with a reactive path that leads to the HO_2_ bound to an oxygen atom of CH_2_OH via a hydrogen bond. This reactive complex has a minimum (M2) along the R_3_ coordinate at 4.05 Å with an energy of 241.4 kJ/mol with respect to the energy of the reactants (see Table S3 of SM). For this molecular adduct, the hydrogen bond has a distance of 1.30 Å, and its associated coordinate is indicated as R_4_. The partial charge of HO_2_ in the M2 complex is almost zero (+0.26e), whereas the CH_2_OH has essentially a unitary charge (+0.74e), as can be seen in fourth panel of Figure 4. The spin distribution of this M2 complex at this point along the MEP shows that the CH_2_OH is almost a perfect singlet, whereas the HO_2_ is a doublet (see third panel of Figure 5). The last panel of Figure 3 shows the MEP along which the M2 complex moves towards the dissociation, leading to the final products CH_2_OH^+^ and HO_2_. Along this dissociation, the spin does not change, while the partial charge varies towards the neutral state for HO_2_ and the +1 charge for CH_2_OH. At an R_4_ of about 2.55 Å the two products already reached their final electronic state.

**Figure 6:**
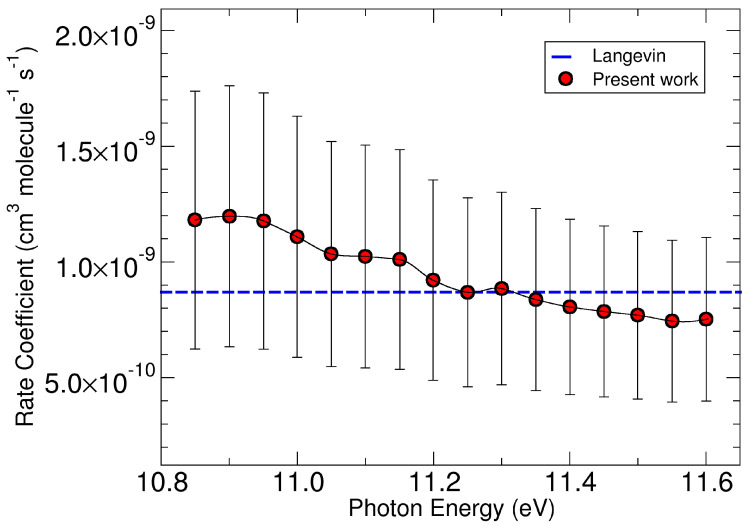
Rate coefficient for the reaction CH_3_OH^+^ + O_2_→ CH_2_OH^+^ + HO_2_ derived from experimental mass data. Further details in the main text and in the SM.

From the above ab initio calculations, it appears that the reactive complex M1, with the following small barrier, is in the region where the reaction slows down. M1 corresponds to a capture complex where negligible intramolecular rearrangements have occurred. This reactive picture should be compared and discussed with the rate coefficient data that can be derived from the experimental data presented in Figure 2. These data have been used to calculate the rate coefficient of the title reaction as a function of the photon energy (see details in SM). In Figure 6, the rate coefficient, which to our knowledge has not been calculated or measured to date, is reported up to a photon energy of 11.6 eV. Data are compared with the rate coefficient calculated with capture theory (Langevin data indicated with blue dashed line in Figure 6). The rate coefficient at the ionization threshold is 1.2 ± 0.6 × 10^−9^ cm^3^molecule^−1^s^−1^, with the Langevin data falling within the experimental rate. The reaction slows down slightly with the increase of h*ν* up to 11.6 eV where the rate coefficient is 7.44 × 10^−10^ cm^3^molecule^−1^s^−1^. This kinetic behavior therefore occurs when the internal energy of the methanol cation increases, i.e., when its vibrational levels are in excited states.

This reaction is therefore particularly fast, also due to the fact that it does not present a reactive energy barrier along the reactive path, but only a small subreactive energy barrier. The bottleneck of the reactive process must therefore be considered in the initial part of the MEP, i.e., at the moment in which the first higher energy reactive complex is formed. This fact indicates that the reaction is essentially governed by long-range forces that determine the formation of a complex according to the capture theory. The subsequent phases of the reaction, i.e., the transfer of hydrogen, the subsequent molecular rearrangement with the formation of the lower energy reactive complex in which there is a hydrogen bond between the protonated formaldehyde and HO_2_, and the final separation into the two products, are all fast processes that do not slow down the reaction.

## 3. Material and Methods

### 3.1. Synchrotron Experiments

The experiments were performed at the Circular Polarization (CiPo) beamline of ELETTRA synchrotron, with the experimental set-up and procedures described in our previous works [36,37,38]. Briefly, the radiation was supplied by an electromagnetic elliptical undulator/wiggler and monochromatized by a Normal Incidence Monochromator (NIM) in the vacuum ultraviolet (VUV) energy range 8–40 eV. The aluminum grating was used to operate only in the energy range of h*ν* = 8–17 eV with a resolving power of about 1000. The photon energy was calibrated against the autoionization features observed in the Ar total photoionization between the 3p spin orbit components. To remove the higher-order radiation contribution at the energy below 11.6 eV, a lithium fluoride (LiF) filter was used. The methanol molecule CH_3_OH/CD_3_OH was introduced in the ion source through a leak valve at a typical pressure of about $10^{-6}$–$10^{-5}$ mbar. The ions produced in the ion source were guided via electrostatic lenses into the octupole (reactive cell) at the nominal collision energy of 0 eV with an energy spread of about 130–150 meV. The neutral reagent O_2_/^18^O_2_ was introduced into the octupole at different nominal pressures from about $10^{-5}$ to 1.2 × $10^{-4}$ mbar and at room temperature. Mass spectra were acquired with the quadrupole before and after the reaction of CH_3_OH^+^ with O_2_ at the photon energy of 11.5 eV in the mass ranges 28 < *m*/*z* < 36 (acquisition time of 4 s/point) while the mass spectra of CD_3_OH^+^ before and after the reaction with O_2_/^18^O_2_ were acquired in the mass ranges of 28 < *m*/*z* < 40 (acquisition time of 10 s/point) always at the photon energy of 11.5 eV. The absence of any signals at *m*/*z* < 28 and *m*/*z* > 40 was also verified before and after the reaction of methanol with oxygen. The intensities of the reagent and product ions obtained from the ion-molecule reactions were acquired in the energy range of 10.8 < h*ν* < 11.6 eV (energy step of 0.05 eV, acquisition time of 30 s/point). CH_3_OH, CD_3_OH and ^18^O_2_ were purchased from Sigma-Aldrich and used at room temperature as received. CH_3_OH has an HPLC gradient grade of 99.93%, CD_3_OH has 99.8% of D atom and a chemical purity of 99%, and ^18^O_2_ has 97% of ^18^O and a chemical purity > 99%. O_2_ was purchased from SIAD with a purity > 99%.

### 3.2. Computational Details

The full geometry optimization of the products, reagents, and bimolecular adducts, as well as the Minimum Energy Path and the harmonic vibrational analysis were performed with the Møller–Plesset expansion truncated at second-order [39,40] using the Gaussian09 software [41]. The basis set used in this work is cc-pVTZ, which is a Dunning’s correlation consistent of basis set at triple zeta [42]. Counterpoise correction to basis set superposition error [43] error was not considered. An estimate of its weight was, however, performed on the M2 molecular complex, and it was found to be 8.7 kJ/mol, or 3.6% of the binding energy of the complex compared to the reagents. This level of calculation gives a reaction enthalpy of 114.2 kJ/mol at 298.15 K, which is about 3% of error with respect to the experimental data of 118.4 ± 10.8 kJ/mol [25]. The spin and charge population were calculated following the Mulliken population analysis [44]. The MEPs were calculated by scanning different interatomic distances while all the other geometrical parameters have always been optimized at each scan point. The scan was carried out with a variable step, and in the regions of the MEP with the greatest slope, a step of 0.05 Å was used.

## 4. Conclusions

In conclusion, our study sheds light on the fast ionic route to hydroperoxyl radical (HO_2_) and protonated formaldehyde formation from methanol radical cation and molecular oxygen, unraveling a previously unexplored reaction pathway in atmospheric chemistry. Through a combination of experimental investigations using synchrotron radiation and computational analysis, we elucidated the dynamics and mechanism of this reaction, revealing the crucial role of hydrogen transfer from the methyl group of methanol to oxygen in generating CH_2_OH^+^ and HO_2_. The rate coefficient is shown to be fast, which indicates that this reactive process is very efficient, and that the hydrogen of the methyl group is transferred to the molecular oxygen along a reaction coordinate that does not present an energy barrier. The study of the behavior of partial charges and spin during the reactive process along the MEP clearly indicates the electronic rearrangement mechanism that governs the dynamics of hydrogen transfer. This electronic rearrangement occurs during the initial moments of the reaction, which is subsequently driven by the intermolecular forces that govern the formation of the reactive complex into the absolute energy minimum and the subsequent evolution towards the final products. Our findings demonstrate the feasibility of a new ion-driven reaction of potential interest in atmospheric processes, particularly in the context of upper tropospheric chemistry where the ionization by cosmic rays is at its maximum [3]. The observed reactivity, characterized by the fast kinetics governed by capture theory, highlights the intricate interplay between ions and neutral species driven by out-of-equilibrium processes induced by cosmic rays. In particular, the spatial, temporal, and non-thermal internal energy distribution of these ions is of great relevance for the outcome of the ion–molecule reactions of atmospheric interest. In this regard, several numerical models have been developed [45,46,47]. However, these models use an average approach, but none of these are made to predict the ionization states of the ions and their spatial distributions. With regard to this, we have recently started to implement interaction models in a Monte Carlo simulation for atmospheric ionization, dealing with O_2_ and N_2_ using Geant4-DNA toolkit [48]. The idea is to expand this study to other important atmospheric molecules such as, for instance, H_2_O, SO_2_, CO_2_, CH_4_, CH_3_OH, NO*_x_*, H_2_O, and O_3_.

By contributing to a deeper understanding of atmospheric processes, our research underscores the necessity for a comprehensive consideration of ion chemistry in climate models, offering valuable insights into the complex interactions driving the Earth’s atmospheric system. Further exploration of ionic pathways and their implications for atmospheric chemistry promises to enhance our predictive capabilities and could inform strategies for mitigating environmental challenges associated with climate change.

## Data Availability

The data presented in this study are available in article and Supplementary Materials.

## Supplementary Materials

The following supporting information can be downloaded at: https://www.mdpi.com/article/10.3390/molecules29071484/s1, Table S1: Cartesian coordinates in Å for the M1B adduct. Table S2: Cartesian coordinates in Å for the M1 adduct. Table S3: Cartesian coordinates in Å for the M2 adduct.
