# Supplementary material for: Ionic Route to Atmospheric Relevant HO2 and Protonated Formaldehyde from Methanol Cation and O2
Source: Molecules. 2024 Mar 27;29(7):1484. doi: 10.3390/molecules29071484 (PMC11013456; doi:10.3390/molecules29071484)
Supplement: Supplementary file 1 [file molecules-29-01484-s001.zip › molecules-2895454-supplementary.pdf]

Supplementary material: Ionic route to atmospheric relevant HO_2 and protonated formaldehyde from methanol cation and O_2

Mauro Satta[1,*], Daniele Catone[2,], Mattea Carmen Castrovilli[3], Francesca Nicolanti[4] and Antonella Cartoni[5,*]

March 20, 2024

[1] Institute for the Study of Nanostructured Materials-CNR (ISMN-CNR), Department of Chemistry, Sapienza University of Rome, P. le Aldo Moro 5, Rome, 00185, Italy; e-mail: mauro.satta@cnr.it
[2] Istituto di Struttura della Materia - CNR (ISM-CNR), Area della Ricerca di Roma 2, Via del Fosso del Cavaliere 100, 00133, Rome, Italy; e-mail: daniele.catone@cnr.it
[3] Istituto di Struttura della Materia - CNR (ISM-CNR), Area della Ricerca di Roma 1, Monterotondo Scalo 00015, Italy; e-mail: matteacarmen.castrovilli@cnr.it
[4] Department of Physics, Sapienza University of Rome, P. le Aldo Moro 5, Rome, 00185, Italy; e-mail: francesca.nicolanti@uniroma1.it
[5] Department of Chemistry, Sapienza University of Rome, P. le Aldo Moro 5, Rome, 00185, Italy; e-mail: antonella.cartoni@uniroma1.it

Correspondence: mauro.satta@cnr.it; Tel.: + 39 06 49913381 (M. S.); antonella.cartoni@uniroma1.it; Tel.: + 39 06 49913678 (A. C.)

Rate Coefficient

In order to obtain the rate coefficient for the title reaction, data from the previous work [1] have been used. In particular we have assumed that for methanol the effective reaction time τ_R, which is a characteristic time interval related to the experimental setup, is the same in the present and previous work. The calculation of the reaction time proceeds by the following scheme. First consider the kinetic equations relating to the bimolecular reaction $A^+ + B \to C^+ + D$, the numerical density of the ionic product N_{C^+} as a function of time is given by:

$$N_{C^+}(t) = N_{A^+}(t=0)\left[1 - e^{-k\cdot N_B t}\right] \tag{S1}$$

where k is the rate coefficient of the reaction, and N_{A+} and N_B are the numerical density of the ionic and neutral reagents respectively. The numerical density of the ionic reagent is given by:

$$N_{A^+}(t) = N_{A^+}(t=0)\cdot e^{-k\cdot N_B t} \tag{S2}$$

Hence the ratio between these two numerical ion densities can be written as:

$$R(t) = \frac{N_{C^+}(t)}{N_{A^+}(t)} = \frac{1 - e^{-k\cdot N_B t}}{e^{-k\cdot N_B t}} = e^{k\cdot N_B t} - 1 \tag{S3}$$

The reaction of the previous work is:

$$CD_3OH^+ + CD_3OH \rightarrow CD_3ODH^+ + CD_2OH \tag{S4}$$

By inversion of eq. S3 the effective reaction time can be derived, having substituted the N_B with N_{CD_3OH}:

$$\tau_R = \frac{ln(1+R)}{k\cdot N_{CD_3OH}} \tag{S5}$$

Here R is the ratio between the mass intensities of the charged product and reagent, N_{CD_3OH} is the numerical density of the neutral gas, which depends on the pressure P, N=P/(k$_B$T).

We have considered data at the Ionization Energy (IE) of 10.84 eV, with P_{CD_3OH}=3.0·10^{-5} mbar, and Mass Intensities (MI): MI(CD_3OH^+)=284±17, MI(CD_3ODH^+)=45±7. The reaction time is calculated to be τ_R=1.7±0.6·10^{-4} s for reaction S4.

For the reaction of the present work we wanted to calculate the rate coefficient by inversion of eq. S5 at the IE=10.8 eV, with P_{O_2}= 1.1·10^{-4} mbar, and τ_R=1.7±0.6·10^{-4} s:

$$k(h\nu) = \frac{ln(1+R(h\nu))}{\tau_R N_{O_2}} \tag{S6}$$

From the mass spectrum at threshold IE we have a mass ratio R=0.70±0.04, and the rate coefficient is k=1.2±0.6·10^{-9} cm^{3}molecule^{-1}s^{-1}. From equation S6, we have calculate the rate coefficients at the different photon energies using the mass ratio at different hν.

Cartesian coordinates of the adducts

	x	y	z
C	-0.030	-0.072	1.425
O	-0.206	-0.042	0.068
O	2.281	-0.008	-1.112
O	2.861	0.041	-2.170
H	0.657	-0.035	-0.430
H	0.951	0.258	1.752
H	-0.902	0.372	1.903
H	-0.132	-1.177	1.632

Table S1: Cartesian coordinates in Å for the M1B adduct.

	x	y	z
C	1.271	0.617	-0.014
O	1.226	-0.708	-0.105
O	-1.312	0.615	0.043
O	-1.414	-0.590	-0.004
H	1.660	1.059	0.901
H	1.472	1.089	-0.975
H	0.042	0.934	0.061
H	1.354	-1.144	0.758

Table S2: Cartesian coordinates in Å for the M1 adduct.

	x	y	z
C	-0.911	0.221	0.418
O	0.098	-0.114	1.045
O	3.076	-0.395	0.756
O	2.229	0.004	-0.118
H	-1.867	0.170	0.931
H	-0.851	0.556	-0.616
H	3.951	-0.356	0.306
H	1.081	-0.051	0.484

Table S3: Cartesian coordinates in Å for the M2 adduct.

References

[1] D. Catone, M. Satta, M. C. Castrovilli, P. Bolognesi, L. Avaldi, and A. Cartoni. Photoionization of methanol: a molecular source for the prebiotic chemistry. *Chemical Physics Letters*, 771:138467, 2021.
